# An Open-Source Wearable System for Real-Time Human Biomechanical Analysis

**DOI:** 10.3390/s25092931

**Published:** 2025-05-06

**Authors:** Zachary Hoegberg, Seth Donahue, Matthew J. Major

**Affiliations:** 1 Department of Physical Medicine & Rehabilitation, Northwestern University, Chicago, IL 60611, USA; zachary.hoegberg@northwestern.edu (Z.H.); seth.donahue@shrinenet.org (S.D.); 2 Jesse Brown VA Medical Center, Chicago, IL 60612, USA; 3 Department of Mechanical and Industrial Engineering, University of Illinois Chicago, Chicago, IL 60607, USA; 4 Shriner’s Children’s Lexington, Lexington, KY 40508, USA; 5 Department of Physical Therapy, University of Kentucky, Lexington, KY 40506, USA; 6 Department of Mechanical Engineering, Northwestern University, Evanston, IL 60208, USA; 7 Department of Biomedical Engineering, Northwestern University, Evanston, IL 60208, USA

**Keywords:** wearable sensors, motion analysis, gait rehabilitation, inertial measurement unit

## Abstract

The advancement of inertial measurement unit (IMU) technology has opened new opportunities for motion analysis, yet its widespread adoption in clinical practice remains constrained by the high costs of proprietary systems, lengthy setup procedures, and the need for specialized expertise. To address these challenges, we present a multi-IMU system designed with streamlined calibration, efficient data processing, and a focus on accessibility for patient-facing applications. Although initially developed for human gait analysis, the modular design of this system enables adaptability across diverse motion tracking scenarios. This work outlines the system’s technical framework, including protocols for data acquisition, derivation of gait variables, and considerations for user-friendly software deployment. We further illustrate its utility by measuring lower-limb gait kinematics in near-real time and providing stride-to-stride biofeedback using a single sensor. These initial results underscore the potential of this system for both laboratory-based gait assessment and rehabilitation interventions in clinical environments and future work will assess validation against traditional optical motion capture methods.

## 1. Introduction

Real-time Biofeedback and Analysis using IMU Tracking (ReBAIT) is an innovative open-source Python 3.9.19 library designed to facilitate kinematic near-real-time visual biofeedback. ReBAIT, as presented in this manuscript, is designed for a gait retraining protocol during treadmill walking but is versatile enough to be extended to other biomechanical applications, e.g., upper limb assessments and tasks, and broader physical therapeutic interventions. This manuscript presents the primary objectives of ReBAIT: (1) to deliver an accessible, real-time biofeedback solution for both researchers and clinicians and (2) to establish a platform that can be extended for gait retraining and other scientific or clinical purposes.

Wearable sensors, particularly inertial measurement units (IMUs), offer a practical solution for enabling real-world biomechanical analysis [1,2,3]. Although methods and models for IMU data collection and analysis are well established, their clinical utility has been hindered by challenges such as integration drift, calibration requirements, magnetic interference, sensor placement variability, and the high costs associated with multi-sensor systems [4]. Furthermore, real-time or near-real-time quantification of movement with minimal setup and computational requirements remains a significant barrier. Despite these known issues, IMUs provide a robust method for directly measuring kinematic variables, such as segmental acceleration, angular velocity, and orientation, rather than deriving these from optical motion capture data [5,6,7]. This can yield more reliable data with less post-processing in situations where a full motion analysis or gait study may be unnecessary or even prohibitive, such as in clinical or home settings [8,9].

**Figure 1:**
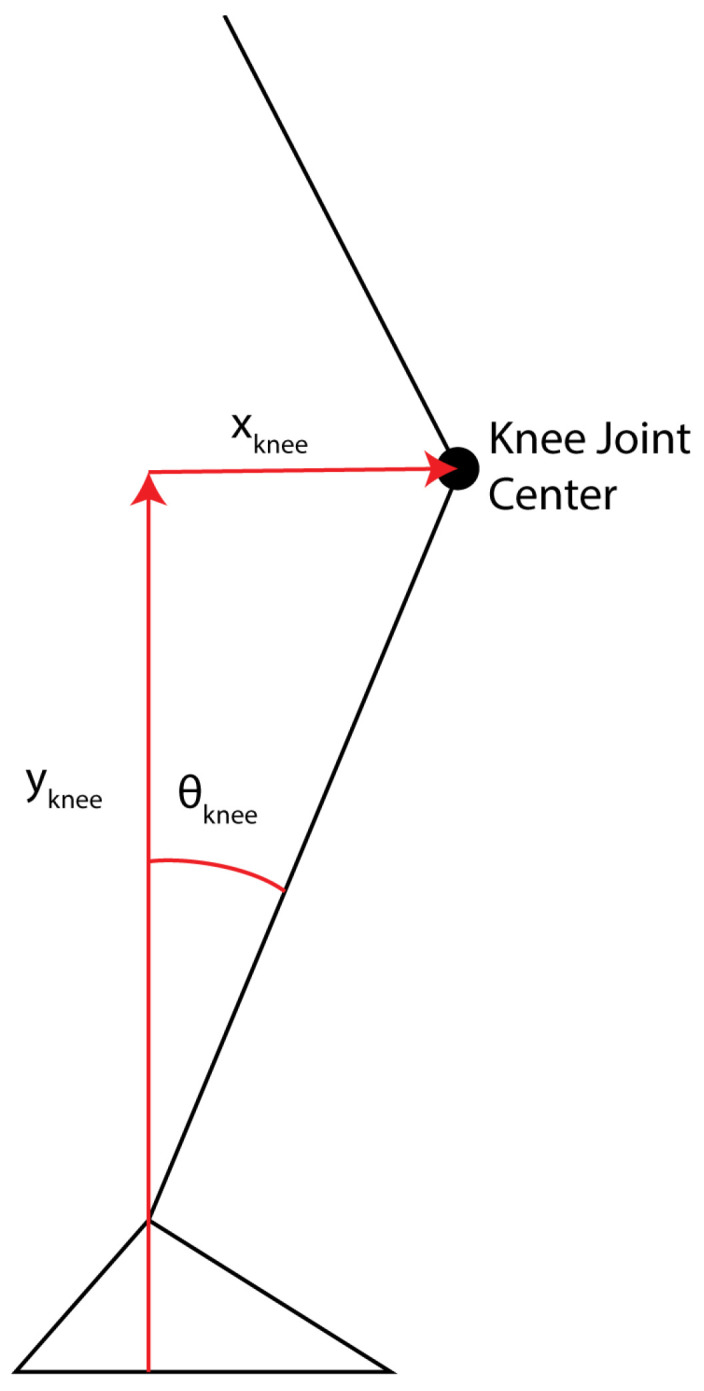
Kinematic components used in the calculation of the LLTE.

In its initial application, ReBAIT computes the lower limb trajectory error (LLTE) from a single IMU and sagittal plane kinematics during treadmill walking at controlled speeds. The LLTE has been defined as the sum of the square of the difference between a measured and reference translation of the knee in the anterior–posterior (A/P) and superior–inferior (S/I) directions and rotation of the shank with respect to the ground during the stance phase, as described by [10,11,12] and shown in Figure 1 and Equation (1). These measurements compare a participant’s lower limb movement against a defined target trajectory, providing actionable biofeedback to meet that target. The long-term vision of ReBAIT is to establish a robust, scalable framework for real-time wearable biofeedback systems applicable to locomotion biomechanics and other domains. This manuscript outlines ReBAIT’s development, with demonstrations of its use for treadmill-based gait analysis, including the calculation of sagittal plane kinematics and LLTE components. While overground biofeedback applications may face practical challenges, as gait and spatial–temporal parameters have been shown to vary significantly [13,14], the ability to quantify gait in real time holds significant value for validating and enhancing clinical observations and monitoring patients in real-world environments beyond spatial–temporal metrics [15,16,17].

The purpose of this manuscript is to provide a tool for gait analysis from a reduced set of IMU sensors for real-time quantification and monitoring of gait. Therefore, this manuscript presents a method for this that begins to address issues with the adoption of IMU-based systems. ReBAIT was designed with the following capabilities:

- Real-time data collection and analysis with Python-based tools;
- Offline data troubleshooting tools that replicate real-time analysis to facilitate debugging and post-processing;
- Algorithms for gait event detection tailored for diverse applications;
- Calculation and visualization of target variables for biofeedback;
- Support for single and multi-IMU configurations to accommodate various research and clinical scenarios; and
- Development of a composite biofeedback variable from a single IMU, consistent with established methodologies.

(1) $$LLTE={\left[\frac{1}{N}\sum_{n=1}^{N}\left\{{\left(\frac{x_{knee,n}^{meas}-x_{knee,n}^{ref}}{leg\;length}\right)}^{2}+{\left(\frac{y_{knee,n}^{meas}-y_{knee,n}^{ref}}{leg\;length}\right)}^{2}+{\left(\frac{\theta_{knee,n}^{meas}-\theta_{knee,n}^{ref}}{\tan^{-1}\frac{foot\;length}{leg\;length}}\right)}^{2}\right\}\right]}^{1/2}$$

## 2. Materials and Methods

### 2.1. Basic Biomechanical Methods

This section provides setup and mathematical details for the biomechanical computations implemented in the ReBAIT library.

- **Sensor Placement:** Section 2.1.1.
- **Calibration:** Section 2.1.2.
- **Joint Angle Calculations:** Section 2.1.3.
- **Lower Limb Trajectory Calculations:** Section 2.1.4.

#### 2.1.1. Sensor Placement and Mounting

**Figure 2:**
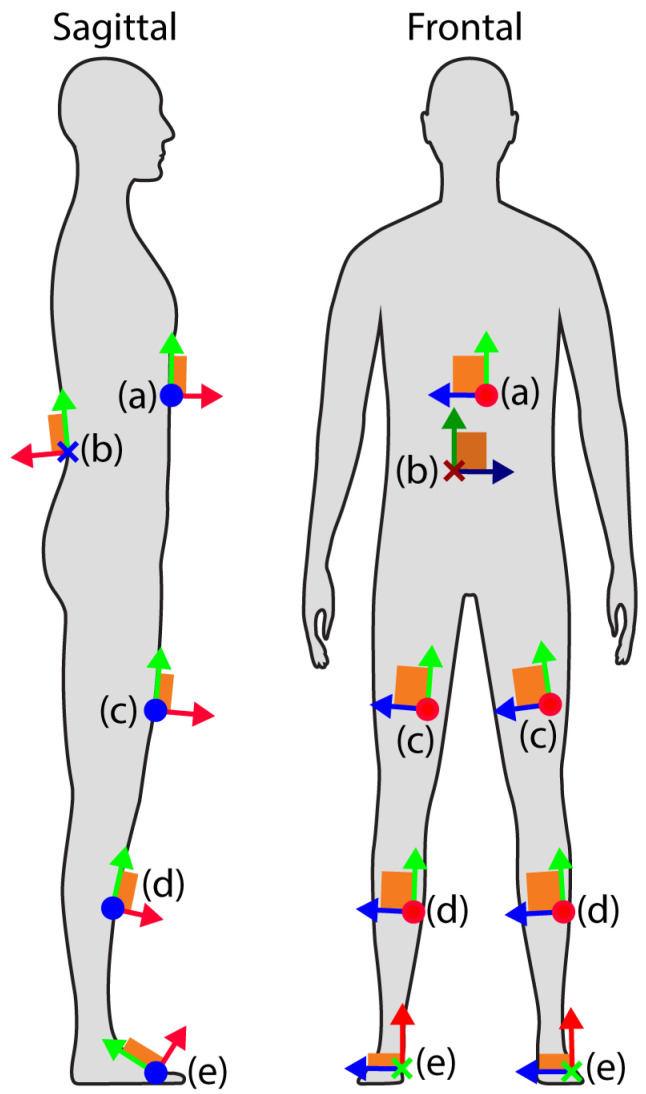
IMU Placement. IMU-fixed coordinate systems are shown, with x-axis (green), y-axis (blue), and z-axis (red). Sternum (**a**): at or slightly superior to the xiphoid process; sacrum (**b**): superior to the midpoint between the posterior superior iliac spine; thigh (**c**): anterior, approximately 15 cm proximal to the knee joint center defined by the midpoint between the femoral condyles; shank (**d**): anterior, approximately 15 cm distal to the knee joint center; foot (**e**): dorsal aspect halfway between the metatarsophalangeal joint and the ankle joint.

Sensors are mounted using hook-and-loop straps on key segments of the lower limb (foot, shank, and thigh), as well as on the sacrum and sternum. Figure 2 illustrates the placement configuration, ensuring consistent alignment for accurate biomechanical data capture.

Once mounted, the distance from the shank IMU to the ipsilateral knee joint center is measured and recorded for calculation of the knee joint position, along with foot length and knee joint height from the floor. Note that these locations can be easily adjusted for other biomechanical analysis needs.

#### 2.1.2. Calibration

Once the IMUs are mounted to the appropriate segments and anthropometric values have been measured, the devices must be calibrated to ensure proper co-orientation of each device. Both a static calibration, consisting of data recording while the subject holds a stationary neutral pose (similar to that depicted in Figure 2), and a dynamic calibration, consisting of data recording during repeated toe-touches and steady-state normal walking, are used to align the IMUs.

Upon completion of the calibration procedure, the data are used to compute the offset between each sensor’s arbitrary global reference coordinate system and a segment-fixed coordinate system consistent with the International Society of Biomechanics (ISB) recommendations [18]. The calibration algorithm has three steps. First, using the linear acceleration data from the static calibration, the direction with the largest magnitude (corresponding to gravity) is aligned with the superior–inferior axis.

(2) $$\hat{n}=\frac{{\vec{g}}_{average}\times {\vec{g}}_{anatomical}}{|{\vec{g}}_{average}\times {\vec{g}}_{anatomical}|}$$

(3) $$\theta =\arccos\frac{{\vec{g}}_{average}\cdot {\vec{g}}_{anatomical}}{|{\vec{g}}_{average}||{\vec{g}}_{anatomical}|}$$

(4) $$q_{g}=\left(\cos\frac{\theta }{2},\sin\frac{\theta }{2}\hat{n}\right)$$

where ${\vec{g}}_{average}$ is the average acceleration vector from the stationary period in the IMU’s global reference frame $I_{G}$, ${\vec{g}}_{anatomical}=9.81\hat{j}$ is the gravitational acceleration vector in the segment-fixed ISB reference frame $S_{i}$, and $q_{g}$ is the quaternion representing the partial transform from $I_{G}$ to $S_{i}$.

Second, a principal component analysis (PCA) is performed on the angular velocity data from the dynamic calibration. The PCA calculates the axis with the largest variation in angular velocity, which corresponds to the axis of rotation for the various segments. This axis is aligned with the medial–lateral axis. The previous work [19] has demonstrated that PCA can robustly determine the knee axis of rotation using an IMU system; we expand that work here to measure hip and ankle joint angles in the sagittal plane.

(5) $$\hat{n}={\vec{e}}_{PCA}\times {\vec{e}}_{1}$$

(6) $$\theta =\arccos\left({\vec{e}}_{PCA}\cdot {\vec{e}}_{1}\right)$$

(7) $$q_{\mathrm{PCA}}=\left(\cos\frac{\theta }{2},\sin\frac{\theta }{2}\mathrm{sgn}\left({\hat{n}}_{y}\right)\right)$$

where ${\vec{e}}_{PCA}$ is the result from PCA analysis of angular velocity, ${\vec{e}}_{1}=\hat{k}$ is the axis normal to the sagittal plane in the ISB reference frame, ${\hat{n}}_{y}$ is the component of $\hat{n}$ in the *y*-direction, i.e., aligned with gravity, and $q_{\mathrm{PCA}}$ is the quaternion representing rotation about the vertical axis to align ${\vec{e}}_{PCA}$ with ${\vec{e}}_{1}$.

Next, angular velocity vectors are rotated to align with a lab-fixed global reference frame $W_{L}$:

(8) $${\vec{\omega }}_{W}=q_{\mathrm{PCA}}q_{g}q_{\mathrm{IMU}}{\vec{\omega }}_{IMU}q_{\mathrm{IMU}}^{\prime }q_{g}^{\prime }q_{\mathrm{PCA}}^{\prime }$$

where ${\vec{\omega }}_{W}$ is the segment angular velocity in $W_{L}$ at a given time step, $q_{\mathrm{IMU}}$ is the IMU orientation quaternion in $I_{G}$ at that time step, ${\vec{\omega }}_{IMU}$ is the segment angular velocity in $I_{G}$ at that time step, and $q^{\prime }$ is the conjugate of quaternion q.

The magnitude of the maximum and minimum angular velocity values are compared, and if the magnitude of the maximum is greater than the magnitude of the minimum, i.e., maxω>|minω|, $q_{\mathrm{PCA}}$ is rotated about the vertical axis by half a rotation to achieve proper alignment of the medial–lateral axis:

(9) $$q_{\mathrm{PCA}}=\hat{j}q_{\mathrm{PCA},0}$$

where $q_{\mathrm{PCA},0}$ is the result of (7).

Once $q_{g}$ and $q_{\mathrm{PCA}}$ are determined, they can be used in conjunction with $q_{\mathrm{IMU}}$ to transform any vector in $I_{G}$ to $S_{i}$, as in (8).

Finally, the average orientation quaternion during the stationary calibration $q_{0}$ is computed for each segment. Right-multiplying by this quaternion will calculate the current orientation quaternion relative to the initial orientation in $S_{i}$:

(10) $$q_{i}=q_{\mathrm{PCA}}q_{g}q_{\mathrm{IMU}}q_{0}$$

When $q_{i}$ is computed for each segment, the rotation between the quaternions for two adjoining segments can be calculated to determine joint angles, as will be discussed in Section 2.1.3.

#### 2.1.3. Sagittal Plane Joint Angles

Joint angles are derived from the orientation quaternions measured by the segment-mounted IMUs. First, each orientation quaternion is transformed using calibration quaternions, aligning the arbitrary IMU reference frame with the fixed laboratory frame and ensuring consistency with segment-fixed frames. This transformation enables the calculation of segment orientations relative to their initial positions in the neutral pose, as recorded during calibration (see Section 2.1.2). The transformed quaternions for each segment are computed using Equation (10).

Once the orientation of each segment is determined, the joint quaternions, representing the relative rotation at a joint between two adjacent segments, are calculated. For example, the knee quaternion is obtained as the rotation between the shank quaternion $q_{\mathrm{SH}}$ and the thigh quaternion $q_{\mathrm{TH}}$. The Equations for hip, knee, and ankle joints are shown in Table 1.

Finally, the joint angles are computed by taking the portion of the joint quaternion that lies in the sagittal plane:

(11) $$\theta_{j}=2\arccos\left(\frac{w_{j}}{\sqrt{w_{j}^{2}+z_{j}^{2}}}\right)\mathrm{sgn}z_{j}$$

where $\theta_{j}$ is the sagittal plane joint angle, and $w_{j}$ and $z_{j}$ are, respectively, the real and $\hat{k}$ components of the joint quaternion $q_{j}$ corresponding to either the hip, knee, or ankle quaternion from Table 1.

#### 2.1.4. Joint Positions and LLTE Corrections

Joint positions are computed by double integration of linear accelerations captured by the IMUs. The position of the knee joint center is an important parameter for calculating the LLTE. Given the well-documented issue of drift during integration, correction methods are essential to ensure accuracy.

**Figure 3:**
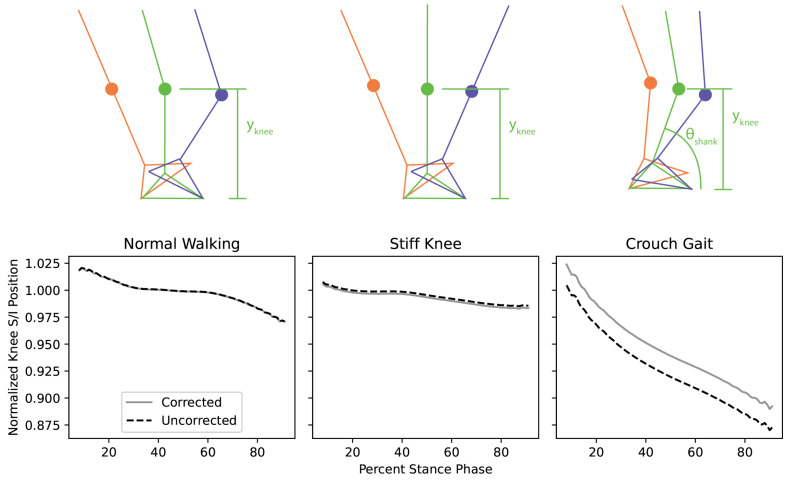
Illustration of corrections to the knee joint S/I position during stance phase for different gait presentations, namely, a stiff or flexed knee gait. The different colors in each of the panels indicate the same time point for each potential walking condition, Orange: Initial Contact, Green: Mid-stance, Blue: Terminal Stance.

Integration is performed only between zero-velocity samples, specifically those closest to mid-stance during each step. Mid-stance and other gait events are determined via an algorithm using the shank angular velocity [20]. Following the approach described in [1], the zero velocity update method limits integration to intervals where accelerations are near zero, reducing drift accumulation. Two key corrections were applied to the knee joint position after integration for the LLTE calculations:

1. Anterior–Posterior Correction: Without correction, the knee A/P position during the stance phase would default to zero due to the absence of a reference frame (Figure 3). This error was resolved by calculating the sine of the shank’s orientation at initial contact and adjusting the initial position to align with that orientation.

2. Superior–Inferior Correction: At mid stance, the vertical position of the knee joint is a known value, as shown in Figure 3. The calculated knee position from double integration was adjusted to enforce this known boundary condition, thereby correcting drift in the superior–inferior direction.

At the end of each gait cycle, the corrected knee position and shank angle from the preceding stance phase are compared to the target trajectory. The LLTE is computed as the deviation from this target, as defined in [12], and scalar value representing the deviation can easily be visualized for the purpose of real-time performance assessment during walking.

### 2.2. ReBAIT Technical Description

This section outlines the software implementation of the biomechanical methods described above and provides guidance on adapting the system for applications beyond gait tracking.

ReBAIT is implemented in Python 3.9.19 and is compatible with most operating systems, though certain restrictions may apply depending on the sensors used or if there are changes to the IMU system used in this study (XSENS, Movella, Henderson, NV, USA). The source code is available on GitHub (https://github.com/sethdonahue73/ReBAIT, (accessed on 29 April 2025)) under the LGPLv3 open-source license. A complete list of dependencies can be found in the requirements.txt file. To assist users, the repository includes Jupyter notebooks detailing the main methods and their usage, with explanations of gait event detection algorithms, references, and illustrative examples. Additionally, short example scripts demonstrate the application of ReBAIT to published datasets, and an example gallery compiles existing methods for quick reference.

At a high level, the system architecture comprises four main components:

- **Callback Class:** Handles incoming sensor data streams, ensuring real-time processing and synchronization.
- **DataCollector Class:** Stores incoming sensor data and performs calculations, including gait metrics and LLTE corrections.
- **PlotFunc Class:** Displays visual biofeedback during data collection, allowing real-time assessment and intervention.
- **ReBAIT Top-Level Function:** Manages and coordinates the interaction between the Callback, DataCollector, and PlotFunc classes, serving as the primary interface for users.

This modular structure enhances flexibility, allowing users to integrate ReBAIT with various hardware and extend its functionality to other biomechanical tracking applications. The design emphasizes ease of use, making it accessible to both researchers and clinicians.

#### 2.2.1. DataCollector Class

**Figure 4:**
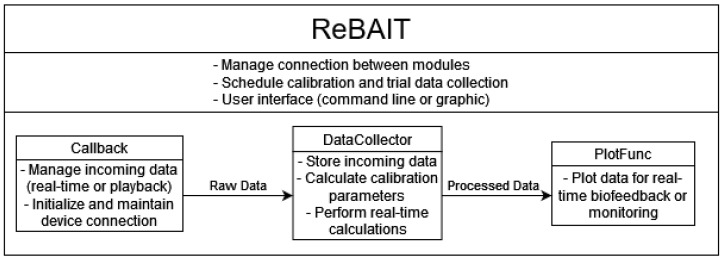
Block diagram of ReBAIT system components and workflow.

The DataCollector class, Figure 4, is responsible for managing incoming IMU data packets, ensuring proper storage, and performing necessary calculations, such as position, gait events, trajectory error (e.g., LLTE), and other relevant kinematic metrics.

Data collection using a DataCollector object runs in a separate thread from the main process. While Python’s global interpreter lock prevents threads from executing on multiple cores simultaneously, this approach improves performance by separating data collection from visualization and processing.

#### 2.2.2. Initialization

When a DataCollector object is created, numpy arrays are pre-allocated to optimize performance during trials. The array size is determined by the IMU sampling frequency and the expected trial duration. For trials exceeding 60 min, modifications to ensure sufficient memory allocation are recommended.

#### 2.2.3. Data Packets

IMUs return data packets at a configurable rate of 100 Hz. Each packet contains the following measurements:

- Timestamp
- Linear Acceleration
- Angular Velocity
- Gravity-compensated Acceleration
- Orientation Quaternion
- Euler Angles

The extracted data are inserted into time-series arrays, maintaining synchronization across all IMUs. To ensure synchronization between IMUs and calculated values, one IMU is designated as the global time reference. For gait analysis, this is typically an IMU used for gait event detection; in this work, the right shank sensor was used as the time reference. This synchronization approach provides robust results even in cases of dropped packets or timing discrepancies between IMUs.

Upon receiving a data packet, values are extracted and appended to their respective arrays. For packets from the global reference IMU, joint angles are computed using the latest orientation quaternions, as described in Section 2.1.3. Gait event detection algorithms are subsequently executed using the most recent angular velocity data. Once a gait cycle is completed, integration algorithms compute limb segment velocities and positions followed by LLTE computation; the LLTE value can then be displayed to the subject as a form of biofeedback.

#### 2.2.4. Visualization

In order to produce visualizations for biofeedback, the calculated LLTE values are plotted in real-time and displayed to the participant. Plotting is achieved using the PyQt5 package, a light-weight library ideal for responsive graphics with minimal latency. The ReBAIT main class accepts an object that extends the QTWidgets.QMainWindow class as an argument. ReBAIT will start the plot when data collection starts after calibration is complete. The supplied object should set up a timer and update function using the QMainWindow framework to automatically update the plot at regular intervals.

### 2.3. Real-Time Data Collection and Processing

The ReBAIT library provides a comprehensive pipeline for real-time IMU data collection, processing, and visualization. This includes initialization, sensor placement, biofeedback generation, and data export, as outlined below.

#### 2.3.1. System Initialization

Before data collection, the XSENS device manager is used to enable sensors, and the Awinda base station and the IMUs must be turned on. Once configured, the manager is closed, and the Python-based system establishes connection with the base station, initializes the IMUs, and displays sensor assignments for mounting.

#### 2.3.2. Script Initialization

Upon running the data collection script, the system initializes IMUs and sets up a data buffer to store incoming packets. The user is prompted to mount the sensors and input anthropometric measurements (e.g., foot length, knee height, shank IMU to knee joint center distance), as described in Section 2.1.1. The target gait pattern is then selected from a library in the case of computing LLTE, and an instance of the DataCollector class is created to integrate sensor callbacks, anthropometric data, and gait patterns.

The ReBAIT system initializes the real-time biofeedback plot using PyQt5. The plotting interface is modular, enabling customization for various research or clinical applications. Visualization updates occur once per gait cycle, ensuring a seamless feedback experience during data collection.

#### 2.3.3. Calibration Process

Calibration proceeds as described in Section 2.1.2. For static calibration, participants assume a neutral pose for five seconds while data are collected. For dynamic calibration, participants perform several toe touches and normal walking steps (typically five to ten of each). Once completed, all IMUs are calibrated for subsequent data collection.

#### 2.3.4. Data Collection and Real-Time Feedback

With calibration complete, data collection can begin. Trials can be conducted continuously or segmented into shorter intervals, with recalibration performed if sensors are bumped or dislocated from their calibrated position. During trials, the system streams real-time data, updates visualizations, and provides biofeedback for participants or clinicians as described in Section 2.2.4. The system’s state is periodically backed up in a temporary file to prevent data loss.

#### 2.3.5. Data Export and File Structure

At the end of data collection, users are prompted to name and save the session data. All raw sensor data and computed variables are compiled into an xarray dataset, aligned by time and category, and saved in a NetCDF (.nc) file. All other values, including trial details and anthropometric measurements, are saved as metadata in the file. Temporary files are deleted upon successful export, and the system allows users to exit or initiate a new session.

### 2.4. Post-Processing and Playback Class

The system includes functionality to “replay” previously recorded sessions for offline testing and development. This feature eliminates the need for a complete sensor setup and calibration, facilitating re-analysis with alternative error functions or targets.

Playback uses the same DataCollector class as real-time data collection but replaces the live sensor callback with a Playback object. This object reads saved data and simulates packet generation, feeding it into the DataCollector for processing. By leveraging saved timestamps, the playback system accurately recreates the original calibration and data collection sequence.

#### 2.4.1. Creating LLTE Reference Targets

Reference (i.e., target) gait profiles for LLTE calculations were generated by collecting data from a pilot participant walking with specific patterns, such as normal unrestricted gait or restricted knee motion using an orthotic brace. Data from 60 s of walking at a set speed were averaged to create normalized knee positions (A/P and S/I components) and shank angles, forming the target reference profiles. These targets were saved as pickle files and can be loaded into the DataCollector for use as real-time biofeedback targets to achieve during gait training, for instance.

#### 2.4.2. DataJoint Post-Processing and Statistical Analysis

Post-processing was performed using DataJoint, a relational database framework for managing computations and analyses. Key tables used in this study include the following:

- **Subject Table**: Contains all unique participants in the study.
- **ImuRecording Table**:
  – **Key**: Participant
  – **Fields**: Project name, list of IMU base files
- **ImuTrial Table**:
  – **Key**: IMU recording and IMU trial ID (a string containing the trial number and condition)
  – **Fields**: IMU file ID (where the trial is located), start and stop indices for each trial, IMU file path, file type
- **ImuTrialInfo Table**:
  – **Keys**: From the IMU trial table
  – **Fields**: Participant’s group number, target for gait training, trial number

The first table should be changed to suit the needs of the user. This table serves as a foundation with primary keys for subsequent tables, such as the following:

- **ImuJointKinematicsNorm Table**:
  – **Keys**: From the IMU trial table
  – **Fields**: Stance phase time normalized sagittal plane joint angles for left and right, hip, knee, and ankle.
- **ImuJointKinematicsSeries**:
  – **Keys**: From the IMU trial table
  – **Fields**: Sagittal plane joint angles for left and right, hip, knee, and ankle from the whole trial.
- **ImuLLTE Table**:
  – **Keys**: From the IMU trial table
  – **Fields**: Target, Trial number, LLTE value, Time normalized LLTE trajectories, Components of the LLTE, and LLTE values from the first and last 30 s of the trial.

From these tables, we extract data for calculating descriptive statistics (e.g., means, standard deviations) for each participant.

### 2.5. Data Collection Protocol

To demonstrate the abilities of our system and validate the utility of the LLTE for measuring deviations in gait pattern, we conducted the study outlined in this section. This study was conducted as part of a larger research project approved by the Northwestern University Institutional Review Board (STU00220488). Twenty-three able-bodied adult participants, who did not have any recent neuromuscular or neurological injuries or chronic impairments (12 F/11 M; age: 26±5 years; height: 1.7±0.1 m; weight: 72.7±17.6 kg), were recruited. Participants were asked to walk at four preset speeds (0.80, 1.00, 1.25, and 1.50 m s^−1^) for one minute each on a treadmill (COSMED USA, Chicago, IL, USA).

Eight XSENS MTw Awinda wireless motion tracker IMUs were initialized and connected to the local Awinda base receiver station. The MTw sensor communicates with the base station using the IEEE 802.15.4 [21] standard and transmits all data at 100 Hz, with sampling time synchronized across all sensors by the base station. Once all the sensors are connected, they are mounted on participants as described in Section 2.1.1 [5]. Anthropometric data, namely, height, mass, knee joint center to floor distance, and foot length, were input into a Python-based system. Following initialization, participants underwent static and dynamic calibration procedures, as described in Section 2.1.2. During trials, participants began walking only after the treadmill reached the preset speed. Data collection started once participants demonstrated steady gait patterns as confirmed visually and continued for one minute. In order to demonstrate the capabilities of this system, we analyzed a subset of data from a larger collection with the participants described above. The purpose of this section and subsequent sections are to provide baseline data and demonstrate the functionality of our system for biofeedback of a single composite variable of lower limb single-plane kinematics.

### 2.6. Data Processing and Statistical Analysis

Data from the lower limb and pelvis IMUs were processed to calculate joint angles and the LLTE variable from the single IMU located on the right shank segment. Joint angle data were gap filled with a cubic join and then filtered with a bi-directional low-pass fourth order Butterworth filter with a cutoff of 24 Hz, following the recommendations of [22], to filter based on the segmental inertial frequencies and not the final kinematic measure. A single walking trial was held out from the 1.00 m s^−1^ due to an error in the data collection process (91 processed trials were included). The LLTE target was generated as the aggregate of the LLTE component trajectories normalized according to the method of [12] during normal walking at 0.80 m s^−1^ across all participants. No filtering or gap-filling were carried out on the calculated LLTE component trajectories. Any stance phase that lasted longer than 1 s was removed from the analysis, as these were outliers due to a missed gait event. A two way repeated measures analysis of variance (ANOVA) assessed the main effects of walking velocity and limb side on stance phase joint range of motion. We expect there to be no differences in the joint angle ranges of motion between the two limbs. In general, we expect the hip joint angle ranges of motion to increase with walking speed, while the knee and ankle remain the same, as has been shown previously with treadmill walking [23]. A one-way repeated measures analysis of variance (ANOVA) assessed the main effect of walking velocity on values of LLTE across participants. Because the LLTE was time normalized to stance phase, we expect there to be no effect of walking velocity on the LLTE values. We also note the effects of each of the constituent components of the LLTE on the overall LLTE value. If there was a significant difference between the dependent values, LLTE or joint range of motion this was addressed post hoc. Statistical analysis was completed with Pingouin Version 0.5.5 Data normality and sphericity were confirmed with the Mauchley test. A Greenhouse–Geisser correction was implemented when sphericity was violated. We report the *p*-value and effect size (partial eta squared, $\eta_{p}^{2}$).

## 3. Results

**Figure 5:**
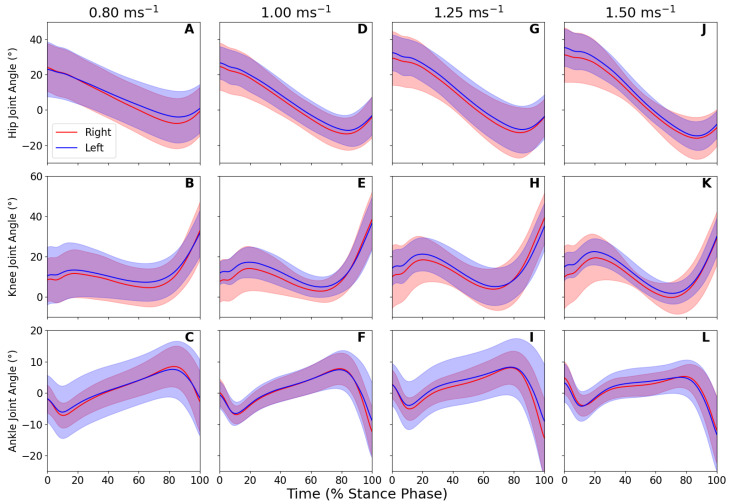
Ensemble averages of time normalized stance phase lower extremity joint angles across participants during normal walking on a treadmill at four different speeds (0.80 m s^−1^ panels (**A**–**C**), 1.00 m s^−1^, panels (**D**–**F**), 1.25 m s^−1^, panels (**G**–**I**) and 1.50 m s^−1^, panels (**J**–**L**)). The solid lines and transparent error bands denote the mean and one standard deviation, respectively.

We first present results showing the measured joint angles during gait and verify that changes in the joint kinematics measured by our system match values from previous work. We then present results of the LLTE measure across speeds and analyze the change in LLTE as gait speed increases. The measured ROMs across a range of walking speeds from 0.80 m s^−1^ to 1.50 m s^−1^ are shown in Figure 5 and Table 2.

The hip joint angle range-of-motion (ROM) during the stance phase was shown to increase as walking speed increases due to increased flexion at initial contact and increased extension at toe off.

The stance phase joint range of motion showed no interaction effects between the walking speed and limb side. Therefore the main effects for each joint were assessed. For hip joint range of motion, the main effect of walking speed was shown to be statistically significant (F=12.28, p<0.05, $\eta_{p}^{2}=0.39$), with no difference in hip range of motion between limbs (F=4.11, p=0.06, $\eta_{p}^{2}=0.18$). Post hoc pairwise comparisons revealed statistically significant differences between hip ranges of motion at 0.80 m s^−1^ and 1.25 m s^−1^ and 1.50 m s^−1^, as well as between 1.00 m s^−1^, 1.25 m s^−1^, and 1.50 m s^−1^, with no statistically significant differences between 0.80 m s^−1^ and 1.00 m s^−1^; and 1.25 and 1.50 m s^−1^. Meanwhile the knee joint range of motion presented no statistically significant differences with respect to speed or limb side: walking speed: (F=1.96, p=0.13, $\eta_{p}^{2}=0.09$), interlimb differences: (F=1.92, p=0.18, $\eta_{p}^{2}=0.09$). Finally, the ankle showed a small increase in dorsiflexion at initial contact and substantially increased plantarflexion at toe off as speed increased. However, these changes were not significantly different as the range of motion was not shown to have any statistically significant differences across speed (F=2.65, p=0.06, $\eta_{p}^{2}=0.12$) or between limbs (F=0.08, p=0.78, $\eta_{p}^{2}<0.01$).

**Figure 6:**
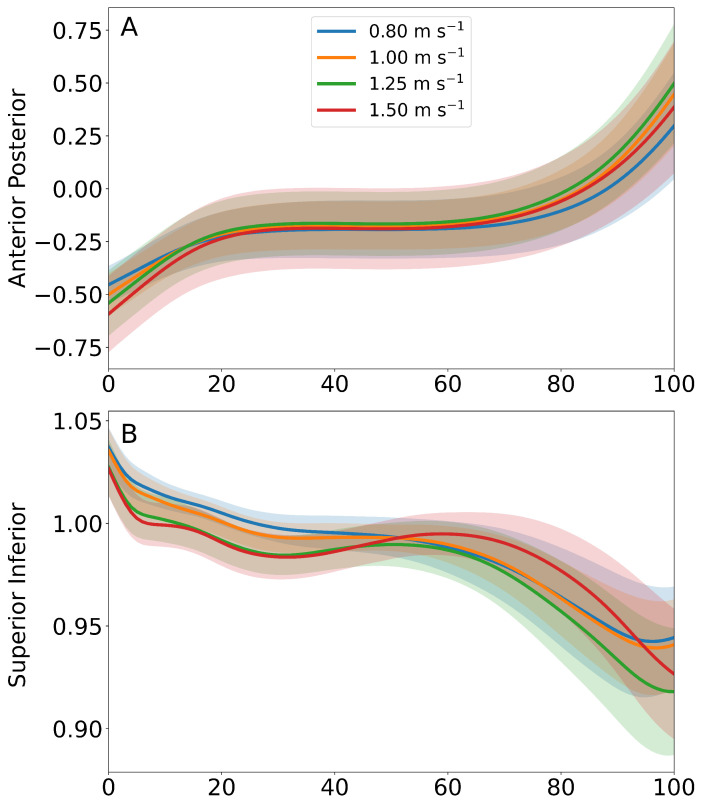

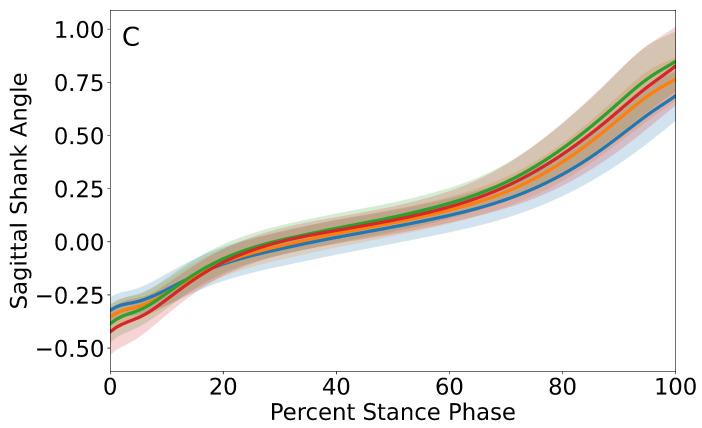
Time normalized A/P knee position (panel (**A**)), S/I knee position (panel (**B**)), and sagittal-plane shank angle (panel (**C**)) trajectories across stance that are components of the LLTE for each walking speed. The solid lines and transparent error bands denote the mean and one standard deviation, respectively.

**Figure 7:**
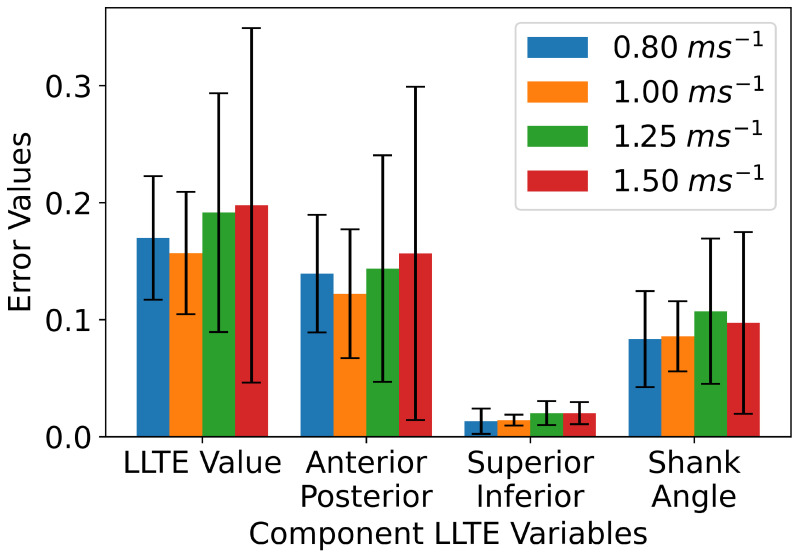
Values of the LLTE and its constituent components across walking speeds when compared to the aggregate 0.80 ms^−1^ across participants as the target trajectory.

During stance phase, the LLTE components appear similar across the range of speeds when normalized to stance time to match the time-normalized LLTE target (Figure 6). The ANOVA results suggested no significant effect of speed on LLTE (F=1.32, p=0.28, $\eta_{p}^{2}=0.04$). These findings suggest that LLTE values are uncorrelated to gait speed changes across the range of tested speeds when using the aggregate 0.80 ms^−1^ as the target trajectory. The contributions of the A/P and S/I knee position and shank angle components to the total LLTE are shown in Figure 7. At each walking speed, the A/P knee position had the greatest effect on the overall LLTE, with the angle swept out by the shank having slightly smaller effect and the S/I shank angle having minimal effect on the LLTE across all tested speeds. It is notable that there are high levels of variability for the A/P error component, which, in turn, dominates the variance of the LLTE values.

## 4. Discussion

We developed ReBAIT as an easy to implement and low cost system that can use only a single sensor to quantify human locomotion and deliver real-time feedback for providing gait training. The accuracy of our system relies on accurate calibration of the IMUs for both joint angles and LLTE calculations. The goal of this work was to introduce the framework and analysis scheme from initial data capture to final outputs and aggregate analysis.

Prior work used an anthropometric model for the estimation of segment lengths and kinematic reconstruction, and a calibration with either specific poses and/or walking forward and backward in their environment [4], while our method utilizes quiet standing in a neutral position and only forward walking. These differences aim to make calibration simple, more accessible, and highly repeatable.

Sagittal-plane joint angles are calculated using the orientation of IMUs on adjacent segments. This method of calculating joint angles mirrors that of a prior work [5] using quaternions in place of rotation matrices; however, our work introduces a novel and straight-forward method of setting up and calibrating the sensors. Measured joint angles during gait approximately match the expected trajectories, and the demonstrated changes in lower limb joint ROM approximately match the previous work analyzing the relationship between gait speed and joint kinematics [24]. The hip joint angle has been measured to increase across walking speed in this study; as walking speed increases, the hip joint modulates stride length, with increased flexion at initial contact and extension before toe off, Figure 5, panels A,D,G,J [24]. Increases in stance phase range of motion at the hip joint have been shown in the previous research. The knee and ankle joint range of motion in the previous research has not been shown to change significantly throughout the stance phase and the gait cycle [14,23], along with walking speed [24].

In a previous work, the LLTE has been limited to use as a simulation and design metric for custom prosthetic feet [11,12,25,26] to encourage gait symmetry in persons with unilateral transtibial amputation. Here, we have extended its use as a biofeedback metric given its unique properties of consolidating multiple kinematic features into a single scalar value. For the purpose of gait training, the two major determinants of the LLTE appear to be the A/P position of the knee and the shank angle swept out during stance phase, while the S/I position of the knee is less variable and therefore more valuable as a design metric for prosthetic ankle–feet (i.e., build height), as shown in Figure 6 and Figure 7. Calculating the LLTE with an IMU requires a number of assumptions and corrections that are not necessary when using marker-based motion capture, as described in the methods in Section 2.1.4. The component trajectories approximately match those presented in the previous work, and the calculated LLTE values are slightly lower than values from the previous studies (0.40 compared to >0.70) [10,11], although those studies compared prosthesis user trajectories to able-bodied.

Furthermore, we have presented evidence that the LLTE values as a stance time normalized metric do not differ significantly with speed. This opens the door to using the LLTE as a biofeedback measure to influence gait for research into motor adaptation or clinical gait retraining within a limited range of speeds.

### Limitations

While the joint angle trajectories appear reasonable and align with the previous work to help verify the platform described here, they have not yet been validated against traditional optical motion capture, gold standard systems; which will be conducted in future work. The calibration method for this system is robust to arbitrary mounting but sensitive to errors or violations of assumptions during the calibration process. For example, we currently assume that during the dynamic calibration period, all of the angular velocity is about the frontal axis. This assumption may not hold exactly in some situations with out-of-plane movement such as hip circumduction and ankle supination/pronation. Additionally, during the static calibration, we assume that the longitudinal axis of the shank and thigh are perfectly aligned with gravity, and this may not always be the case, especially in certain patient groups with large skeletal morphological deviations. We note that the large effect sizes in the range of motion data without statistically significant differences may indicate high variability in the ranges of motion measurements, other numerical methods might be considered to reduce this variability. Our platform was assessed in a controlled environment with fixed walking on a treadmill with motion primarily limited to the sagittal plane, and therefore, future work should involve assessment in more dynamic scenarios and validation against the traditional marker-based motion capture, and this is a known limitation of the system. Finally, our system is subject to common issues with IMUs such as drift and offsets, which are highly dependent on the quality of the specific sensor used. Our system currently makes no attempt to measure and calibrate out any sensor bias, instead relying on the sensor’s internal filters. For this work, the Xsens MTw IMU was used, which has an angular velocity bias stability of 10 deg/h and an acceleration bias stability of 0.1 mg, as well as an orientation accuracy of ≤1.5 deg RMS [27]. Future work plans to look at the behavior of the system with other IMUs, including common low-cost IMUs that may further increase accessibility of motion tracking.

## 5. Conclusions

In conclusion, we have developed and demonstrated the capabilities of a simple-to-use IMU system for the estimation of lower limb kinematics that allows for data visualization for near-real-time biofeedback from single or multiple sensors. Our system offers advantages in terms of applicability to researchers and clinicians due to minimal setup, operation, and technical expertise compared to more traditional motion capture systems by the use of a subset of sensors on specific limbs instead of the whole body, leading to the ability to be used in near-real time for quantification of kinematics and simplicity of the calibration process. Planned future work on this system includes expanding applicability to track upper limb kinematics, tracking during overground walking, and additional joint angle measurements that include transverse and frontal plane angles.

## Figures and Tables

**Table 1:** Lower Limb Joint Rotation Quaternions.

Hip	$q_{h}=q_{\mathrm{PV}}^{\prime }q_{\mathrm{TH}}$
Knee	$q_{k}=q_{\mathrm{SH}}^{\prime }q_{\mathrm{TH}}$
Ankle	$q_{a}=q_{\mathrm{SH}}^{\prime }q_{\mathrm{FT}}$

**Table 2:** Mean sagittal plane joint range of motion (mean ± SD) for each lower limb joint across participants, plotted by walking speed. Significant pairwise differences from 0.80 m·s^−1^ are indicated with * and those from 1.00 m·s^−1^ are indicated with †.

Joint	0.80 m s^−1^	1.00 m s^−1^	1.25 m s^−1^	1.50 m s^−1^
Right Hip	39.18 ± 11.84	42.68 ± 7.75	48.55 ± 8.64 *^,†^	55.13 ± 7.32 *^,†^
Right Knee	38.40 ± 13.57	41.79 ± 10.30	42.82 ± 16.35	39.85 ± 17.15
Right Ankle	28.63 ± 29.60	25.34 ± 6.99	27.72 ± 8.20	23.11 ± 8.87
Left Hip	34.44 ± 7.34	41.23 ± 8.28	46.91 ± 10.40 *^,†^	54.17 ± 12.79 *^,†^
Left Knee	33.00 ± 7.99	37.52 ± 10.62	36.24 ± 10.41	36.20 ± 8.02
Left Ankle	22.28 ± 4.71	24.13 ± 7.49	25.52 ± 9.09	24.87 ± 10.20

## Data Availability

https://www.openicpsr.org/openicpsr/project/212941/version/V1/view (accessed on 20 March 2025).

## Abbreviations

The following abbreviations are used in this manuscript:

ReBAIT	Real-time Biofeedback and Analysis using IMU Tracking
IMU	Inertial measurement unit
ISB	International Society of Biomechanics
PCA	Principal component analysis
LLTE	Lower limb trajectory error
ANOVA	Analysis of variance
ROM	Range of motion
A/P	Anterior–posterior
S/I	Superior–inferior
